# Mediterranean seagrasses provide essential coastal protection under climate change

**DOI:** 10.1038/s41598-024-81026-5

**Published:** 2024-12-04

**Authors:** M. Agulles, N. Marbà, C. M. Duarte, G. Jordà

**Affiliations:** 1 Instituto Español de Oceanografía, Centro Oceanográfico de Baleares (IEO-CSIC), Palma, Spain; 2 Mediterranean Institute for Advanced Studies (CSIC-UIB), Majorca, Spain; 3 Red Sea Research Centre (RSRC) and Computational Bioscience Research Center (CBRC), King Abdullah University of Science and Technology, 23955 Thuwal, Saudi Arabia

**Keywords:** Physical oceanography, Environmental impact

## Abstract

Seagrasses are vital in coastal areas, offering crucial ecosystem services and playing a relevant role in coastal protection. The decrease in the density of Mediterranean seagrasses over recent decades, due to warming and anthropogenic stressors, may imply a serious environmental threat. Here we quantify the role of coastal impact reduction induced by seagrass presence under present and future climate. We focus in the Balearic Islands, a representative and well monitored region in the Mediterranean. Our results quantify how important the presence of seagrasses is for coastal protection. The complete loss of seagrasses would lead to an extreme water level (eTWL) increase comparable to the projected sea level rise (SLR) at the end of the century under the high end scenario of greenhouse gases emissions. Under that scenario, the eTWL could increase up to ~ 1.4 m, with 54% of that increase attributed to seagrass loss. These findings underscore the importance of seagrass conservation for coastal protection.

## Introduction

Coastal areas are a vital system for a significant proportion of the global population and economy^1^. However, they are highly susceptible to the effects of climate change, with rising sea levels, increased storm surge flooding and wave impacts threatening human lives and coastal infrastructures^2,3^. Traditional solutions to coastal protection from flooding, achieved through “hard engineering solutions” (e.g., seawalls, dikes), provide limited protection and involve long-term environmental impacts. Hence, nature-based options, which incorporate ecology and ecosystem services to achieve coastal protection, is gaining prominence^4^alone or in a hybrid model combined with “hard engineering solutions”^5^. The presence of submerged vegetation can be an effective way to reduce the energy of the incoming waves and, thus, to minimize the impacts of marine storms as elements of coastal protection^6^.

*Posidonia oceanica* is an endemic Mediterranean marine angiosperm that forms extensive, dense and highly productive submerged seagrass meadows^7^. In the Mediterranean Sea, where about 1/3^rd^of the population of the surrounding countries live in the coastal zone^8^, *P. oceanica* meadows cover 22,984 km^2^ of the coastal areas, or about 25% of the sea bottom between 0 and 35 m depth. *P. oceanica* meadows play a key role in supporting Mediterranean fisheries production, carbon sequestration, and increase sediment retention which, therefore, reduces the erosion in the coastal zone^9,10^. Consequently, seagrasses are an extremely important component of the coastal ecosystems^7,^ and their distribution and abundance reflect coastal environmental quality^9^.

Seagrass meadows interact with flows, reducing the significant wave height by about 20–30%, as demonstrated in both laboratory flume experiments^11,12^and field experiments^13,14^. Nevertheless, most assessments of the role of seagrass meadows on the reduction of coastal impacts are local in nature and studies at higher spatial scales are still lacking, precluding incorporating seagrass into regional nature-based or hybrid solutions for coastal protection under climate change. The focus on local scales is due to the lack of adequate numerical modelling to generate regional-scale estimates. Fortunately, numerical tools have been developed to simulate wave propagation over benthic vegetation, including realistic models that resolve the 3D Navier–stokes equations and include the representation of wave-vegetation interactions^15,16^. In addition, an analytical model for wave transformation over vegetation fields based on a non-linear formulation of the drag force was presented by^17^and subsequently implemented in the vegetation module of the nearshore wave propagation numerical model named XBEACH^18^, in order to simulate wave propagation over seagrass meadows under different configurations^16^.

**Fig. 1:**
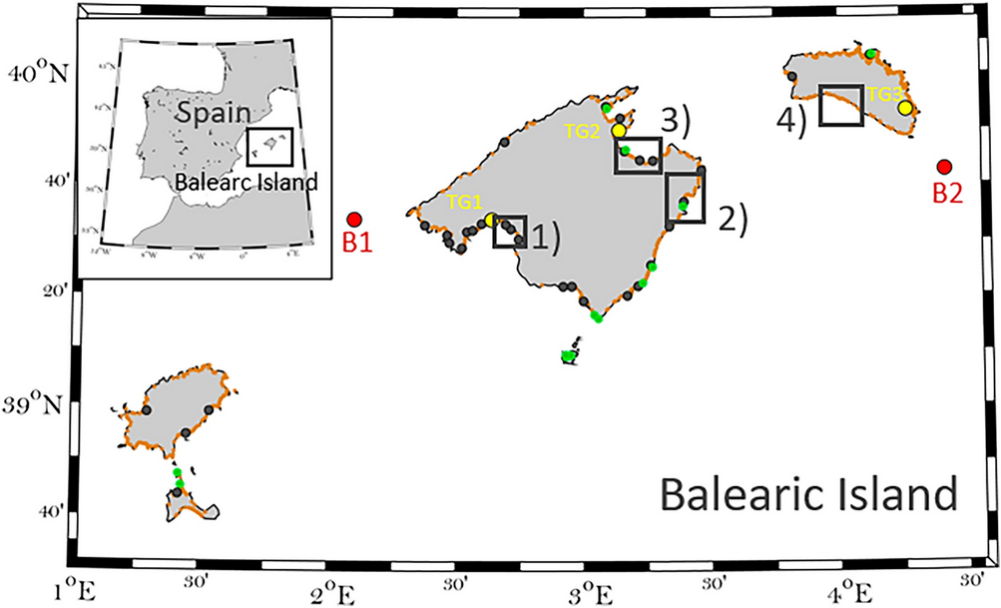
Map of the study region including in-situ observations of shoot density of P. oceanica (green dots^9^,), beach locations (orange dots) and port locations (black dots). The sites selected for the analysis of the evolution of seagrass meadow shallowest front are indicated by the black squares (1-Platja de Palma, PDP, 2- Cala Millor beach, CLM, 3- Badia d’Alcudia, BDA, and 4-Son Bou beach SNB). Red dots denote the buoys locations for offshore waves validation (B1-Sa Dragonera and B2-Mahón). The Tide gauges used for sea level validation are located in yellow dots from Palma Port (TG1), Alcudia Port (TG2) and Mahón Port (TG3). Figure created by M_Map tool, Matlab version 2022b (https://es.mathworks.com/products/new_products/release2022b.html).

The role of seagrasses in coastal protection has been, however, compromised due to losses in seagrass extent derived from coastal development and deteriorated water quality^19^, as well as, more recently, the increase of marine heatwaves due to climate change, to which *P. oceanica* is particularly vulnerable^20,21^. Losses of *P. oceanica* abundance in the Mediterranean sea are estimated at about 6.9% per year over the last 50 years due to several causes like pollution, mechanical stress and recently, the spread of invasive exotic species^22^, although lost this rate has decelerated since the 1990s^9^. Moreover, future projections suggest that warming could lead to the functional extinction of *P. oceanica* meadows during the second half of this century^23–25^. The impact of past and future seagrass losses on coastal protection as sea level rises remains, however, unassessed. Here we quantify the role of seagrass in reducing the total water level (TWL) at the coast during extreme events, as they are responsible for coastal flooding and erosion, under present and future climate. We focus on the Balearic Island (Fig. 1), a well monitored region where the economy is strongly dependent on beach tourism.

### Evolution of the seagrass front

**Fig. 2:**
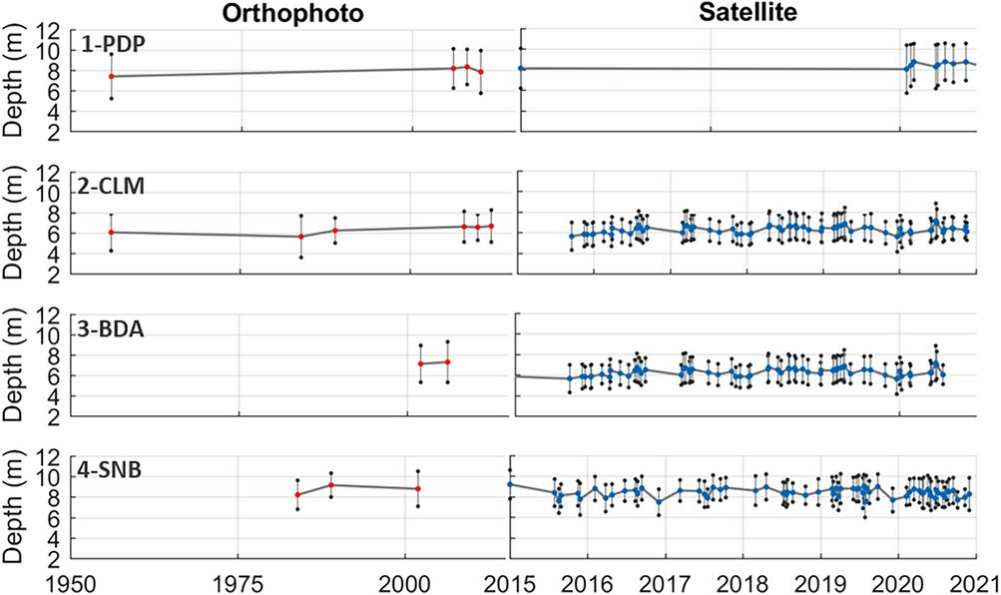
Average depth of seagrass front for; 1) Platja de Palma (PDP), 2) Cala Millor (CLM), 3) Badia d’Alcúdia (BDA) and 4) Son Bou (SNB). In the left panel, results of depth average (red dots) and STD (vertical gray lines) retrieved from orthophoto images and in the right panel, results of depth average (blue dots) and STD (vertical gray lines) retrieved from satellite images. Note that the scale of the horizontal axis is different among left and right panels.

To evaluate the changes in the position of the shallowest edge of the seagrass (referred to as the seagrass front) over time, ortophotos and satellite imagery were analyzed (see methods section) for four different study locations (black squares in Fig. 1). The analysis of the images reveals that the water depth at the upper limit of the meadow remained practically constant over the time period examined (1956–2021) at the sites examined, except Playa de Palma (Fig. 2). In Playa de Palma (PDP), the seagrass meadow starts at 8.31 ± 0.38 m depth. At Son Bou (SNB) it is located at 8.34 m depth but exhibits more variability along the years (± 0.57 m). In Cala Millor (CLM) and Badia d’Alcudia (BDA), the upper edge of the meadow located at 6.28 ± 0.39 m and 7.17 ± 0.41 m, respectively.

The orthophotos show a regression of the PDP seagrass upper front from 7.4 to 8.1 m in 58 years (1956–2014), which the satellite images further extend to 8.8 m recently (2020–2021). However, this must be taken with caution because observations are not uniformly distributed across the time period examined (Fig. 2a). In CLM, the orthophotos suggest a recession of the seagrass upper limit from 6.0 m to 6.8 m from 1959 to 2021. However, the satellite images show a very variable depth ranging from 5.3 to 7.2 m in the last 6 years. This suggests that there is a non-negligible level of observational noise (i.e., maybe due to dead leaves remaining in the bottom interpreted as seagrass) that probably also affected the values obtained from the orthophotos. The same behavior is observed in BDA and SNB from the satellite images (the record from the orthophotos in those locations is too short to be considered). In summary, we have not found consistent evidence for a long-term regression of the seagrass shallow limit, except in the case of PDP. Therefore, the shallow limit of the meadow was kept constant in the simulations performed.

## Role of seagrass for coastal protection in present climate

**Fig. 3:**
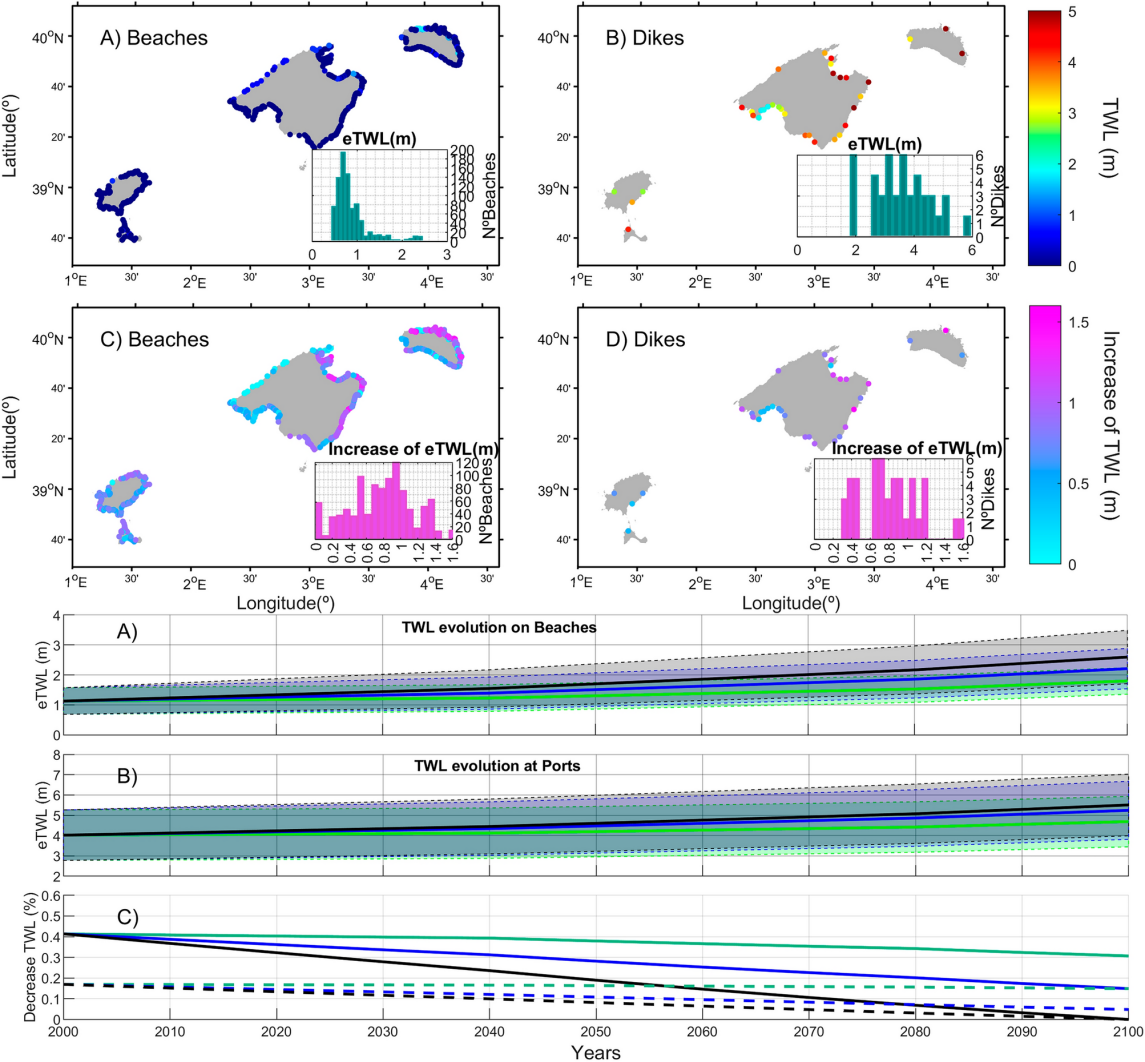
**(A**-**B)** Simulated 99^th^ percentile of the total water level in the Balearic Islands (experiment PRES_WS). The results are presented for the beaches (a) and the dikes (b). (**C**-**D)** increase of extreme TWL in the absence of seagrasses (difference between experiment PRES_NS and experiment PRES_WS). The results are presented for the beaches (c) and the dikes (d). For all panels, the histograms of the values mapped are presented in the inlays. E–F: Average evolution of extreme TWL in the Balearic Islands beaches (E) and ports (F) under RCP8.5 GHG scenario and under different scenarios for the evolution of seagrass meadows. No degradation (green lines, simulation FUT_615), moderate degradation (blue lines, simulation FUT_200) and total degradation (black lines, simulation FUT_NS). Patches represent the spatial Standard Deviation (STD) of the extreme TWL. The bottom panel, (G) represents the contribution of P. oceanica to reduce the eTWL in percentage (%) with respect NS scenario in beaches (continuous lines) and ports (dashed lines). Figure created by M_Map tool, Matlab version 2022b (https://es.mathworks.com/products/new_products/release2022b.html).

The TWL at 3 hourly resolution is considered as the sum of the mean sea level, the storm surge, and the wave runup and was obtained along the coast every 200 m (see methods section). Extreme total water level (eTWL), defined as the 99% quantile of the historical TWL, is higher at the dikes than at the beaches (Fig. 3, A-B panels). The reason is twofold. First, the dikes are located at deeper depths where the wave height is higher and, second, their shape favors higher wave runup. The values of the extreme TWL in the beaches range from 0.45 m in the southern coast to 2.45 m in the northwest of Mallorca and northern coast of Menorca with an average value of 0.87 ± 0.39 m, depending on the different orientation of the beaches relative to the incoming storms, the beach slopes and the seagrass coverage. In the ports, the TWL ranges from 1.80 m in the Palma Bay to 6.67 m in northern coast of Mallorca and Menorca, with an averaged value of 3.74 ± 1.2 m. In addition to the previously mentioned factors, the type of dike also plays a role in TWL with the vertical walls inducing higher runups in the ports.

Loss of *P. oceanica* meadows lead, on average, to an increase of about 0.70 m in the extreme TWL both on beaches and dikes, with larger increases (> 1.2 m) on the Northern coast of Menorca and Mallorca (Fig. 3, C-D pannels). Those larger increases (> 1.2 m) would be felt in 77 beaches (4 ports) out of the total 869 beaches (34 ports) in the region. Conversely, extreme TWL is less vulnerable to seagrass loss in the beaches of the southern part of Menorca and beaches and ports in the Palma Bay, where the expected TWL increase with seagrass loss is estimated to be about 0.30 m, affecting 130 beaches and 8 dikes.

## Role of seagrasses for coastal protection under future scenarios

To develop future scenarios of coastal total water level (TWL), it is essential to have future projections of sea level, wave patterns, and seagrass characteristics. For the two first elements we use published results (see Methods sections). For seagrass characteristics projections , three potential scenarios for seagrass evolution are considered based on the observed seagrass density: (1) the meadows remain stable, (2) the meadows completely dissapear by the end of the century due to global warming, and (3) the meadows follow a middle-ground scenario where they gradually decline from 615 shoots/m^2^ to 200 shoots/m^2^ by the end of the century.

If seagrass meadows are conserved, projected mean sea level rise would lead to the extreme TWL in beaches to go from 1.07 m (on average) to 1.74 m, and from 3.96 m to 4.63 m in ports, at the end of the century. Seagrass decline is estimated to have a large effect on the extreme TWL, with moderate degradation of the meadow (current density declining by 2/3’s, i.e. down to 200 shoots m^−2^ at the end of the century) leading to an increase of the extreme TWL in beaches of 1.08 m, on average, increasing from the present 1.07 m to 2.15 m (Fig. 3e, blue). In ports, TWL is projected to increase from 3.96 up to 5.18 m under the same scenario (Fig. 3f, blue). Complete seagrass loss would lead to TWL increasing by 1.46 m, on average, on beaches (Fig. 3e, black), 0.79 m of this attributable to seagrass loss, while TWL will increase by 1.49 m on ports (Fig. 3f, black), of which 0.82 m are attributable to seagrass loss (Table 1). Thus, conserving seagrass meadows reduces the projected increase in extreme TWL on beaches by an average of 40% under present conditions and by 30% at the end of the century, compared to Balearic shorelines devoid of seagrass meadows. Under present conditions, the seagrass reduces the extreme TWL in dikes by 18% (see Fig. 3d). There is, however, broad variability in these projections, attributable to local characteristics (Table 1). This means that in some locations, the loss of seagrass can induce an increase in the extreme TWL of up to 2.35 m. Sensitivity analyses show that wave dissipation is greater when seagrass meadows reach shallow depths, have long leaves, wide stems or high shoot density, both for moderate and strong storms. These seagrass traits have similar contributions to energy dissipation, except for the extreme case that seagrass grow to 1 m depth (Extended Data Fig. 8).

**Table 1:** Average extreme TWL in beaches and ports (in meters) under different scenarios of seagrass evolution under present and future conditions.

Run	Seagrass degradation in 2100	Average present eTWL (in m)	Average Future eTWL (in m)	Projected Change (in m)	Contribution of MSLR to eTWL increase in 2100 (in %)	Contribution of seagrass loss to eTWL increase in 2100 (in %)
BEACHES						
FUT_NS	Total	1.07 (0.49–2.81)	2.54 (1.50–3.97)	1.46 (0.64–2.35)	46	54
FUT_200	Moderate (200 shoots /m^2^)	1.07 (0.49–2.81)	2.15 (1.35–3.48)	1.08 (0.67–1.64)	62	38
FUT_615	No degradation (615 shoots/m^2^)	1.07 (0.49–2.81)	1.74 (1.16–3.48)	0.67	100	0
PORTS						
FUT_NS	Total	3.96 (1.93–6.99)	5.45 (2.99–9.29)	1.49 (0.96–2.30)	45	55
FUT_200	Moderate (200 shoots /m^2^)	3.96 (1.93–6.99)	5.18 (2.87–8.77)	1.22 (0.87–1.77)	55	45
FUT_615	No degradation (615 shoots/m^2^)	3.96 (1.93–6.99)	4.63 (2.59–7.66)	0.67	100	0

The range of values in the region is presented in brackets (min–max). Last two columns show the relative contribution of MSLR and seagrass loss to the projected changes in 2100 (in %).

**Fig. 4:**
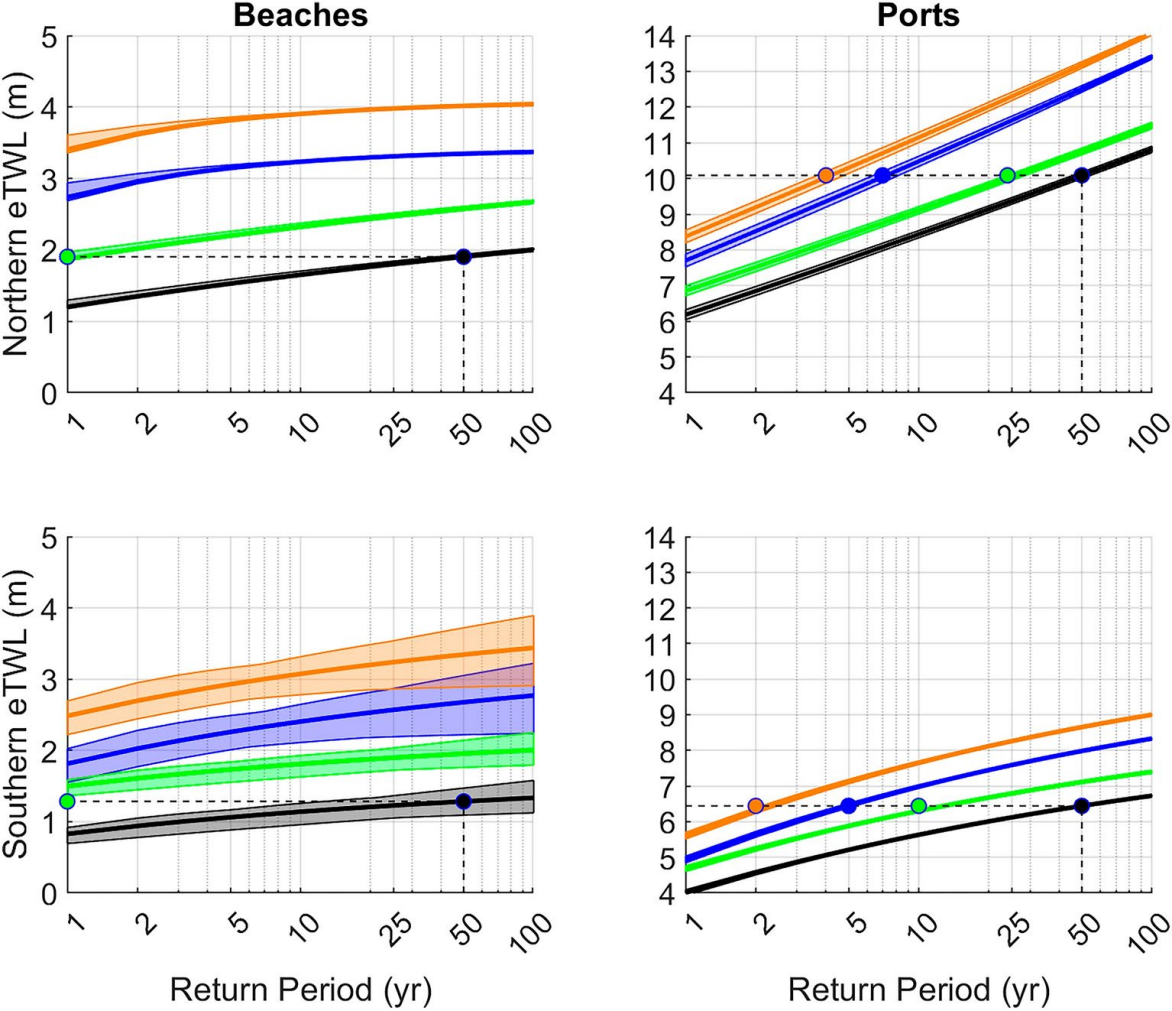
Return Levels on beaches (left panels) and ports (right panels), in the northern (top panels) and the southern (bottom panels) coasts of Mallorca, for different scenarios of seagrass conservation and different time frames. Present conditions (black), present conditions with seagrass absence (blue), 2100 conditions with well-preserved seagrasses (green), and 2100 conditions with seagrass absence (orange). Note the vertical axis in Beaches and Ports differ. The dotted line indicates an extreme total water level with a return period of 50 years under present climate. The colored dots indicate at which period would that storm correspond in the different scenarios.

The return levels (RL), the magnitude of the events associated with a given return period (average time interval between occurrences of such event), provide additional insights into the role of seagrass on coastal protection. In our case, RL are computed using the peaks over threshold approach^26^associated to extreme events (Extended Data Fig. 7). An extreme TWL associated to a RL of 50 years, as could be the case of the Gloria storm that hit the Spanish Mediterranean in January 2020^27^, could reach a value of about 2 m in the northern beaches and 10 m on the northern dikes (Fig. 4a, b black dots). In the south of Mallorca Island, the RLs are lower, being ~ 1.2 m and ~ 6.5 m for beaches and dikes, respectively. Under present marine conditions but in the absence of seagrass meadows, one in 50 years storm like Gloria would have induced a higher TWL reaching 3.3 m and 12.5 m in northern beaches and dikes (Fig. 4a,b blue dots) and 2.5 m and 8.0 m in the southern beaches and dikes (blue lines in Fig. 7c). Moreover, the impacts of such storms would be felt much more often: every year in beaches and every 5 years in dikes.

Assuming seagrass meadows to be conserved (orange curve in Fig. 4), the storm-induced impacts that occur every 50 years on average at present will occur in the northern and southern dikes of the region every 23 and 12 years, respectively. On beaches, the effects of the sea level rise would be even more acute, and the impacts associated to any storm would be similar to those occurred with the extreme Gloria storm. Loss of seagrass meadows and sea level rise will expose the northern and southern coasts of Mallorca to Gloria-type impacts every 3 and 2 years, respectively and the extreme TWL on the beaches would be very high, reaching 4 and 3.3 m every year, respectively, much higher than what was experienced during the Gloria storm.

## Discussion

Seagrass meadows play a significant role in wave energy dissipation and, consequently, in the risk and intensity of coastal flooding during storms. However, the role of seagrass conservation in protecting shorelines under climate change has not yet been quantified. Consequently, the estimates presented here provide a necessary foundation linking conservation and coastal adaptation planning.

Specifically, our model calculations show that seagrass reduces the incoming wave height by 20–30%, at the position where the ratio (depth/Hrms) is lowest (Extended Data Fig. 9, sensor P4), in comparison to bare seabed, consistent with results from flume experiments^11,12,28^. Similar seagrass effects have been observed in field experiments, with wave height being reduced by 25–49% when compared to adjacent bare seabeds^14^. Seagrass effects on wave damping are more intense when incoming wave energy is higher (i.e., under storm conditions), while the presence of seagrasses does not change significantly the TWL under calm conditions (Extended Data Fig. 6). The effectiveness of the role of seagrass in attenuating wave action is also higher at shallower depths than at deeper ones, thus meadows with the upper limit located closer to the shore reduce the wave energy more effectively. Conversely, the effect of the seagrass is negligible at depths approaching the depth limit of *P. oceanica*meadows (30–40 m^29^,). The effect of seagrass on wave reduction is larger at locations with gentle slopes, as the wave-seagrass interaction lasts longer. Therefore, seagrass can reduce the extreme wave runup between 10 and 60% at beaches and between 10 and 20% in ports across the Balearic Islands, depending on the level of exposure and seagrass characteristics.

Our analyses assumes that the seabed becomes sandy when seagrass dies (i.e., bottom friction coefficient of 0.02), which is an oversimplification, since following plant death the roots and rhizomes remain in the ground for a certain time forming a seabed structure known as “matte” that acts as a rocky base in terms of friction (Manning bottom friction coefficient of 0.030–0.035). Although there is not enough information for that process to be more accurately incorporated in our scenarios, we estimated that if rhizomes of dead plants would form a ”matte” seabed, the wave runup during extreme events could be reduced 5–10%, which has little impact on our findings.

Our assessment focused on a single emissions scenario (RCP8.5) for simplicity. Under GHG scenario RCP4.5 the mean sea level rise by 2100 is estimated to be ~ 0.50 m^30,31^ and therefore the extreme TWL at the end of the century would be smaller. However, *P. oceanica* meadows are expected to play a key role in the coastal protection under any GHG emission scenario, with their contribution to reduce extreme TWL ranging from ~ 30% (in the worse sea level rise scenario) to ~ 40% (considering no change with respect to present sea level, see Fig. 3).

Our results show that the loss of the seagrasses would have an impact comparable to that of the projected impact of mean sea level rise, thereby doubling the impact of climate change on Balearic coastal areas. For the beaches^32^, showed that the projected mean sea level rise would imply the loss of 86% of beach area in the Balearic Islands under storm conditions. This is similar to the effect of seagrass losses alone on beach erosion.

The dikes and seawalls of ports are typically designed to resist events with a return period of 50 years. Thus, dikes and seawalls should be re-designed, and seagrass conservation and restoration should become a priority in coastal defense plans in order to adapt to sea level rise. If coastal protection against projected sea level rise is based exclusively on hard coastal defense structures, dikes and seawalls should be re-designed to be even greater. The inclusion of seagrass meadows in coastal defense, in addition, would provide co-benefits beyond coastal protection such as mitigation of climate change, biodiversity and good water quality. Sensitivity analyses show that wave dissipation is greater when seagrass meadows reach shallow depths, have long leaves, wide stems or high shoot density, both for moderate and strong storms. These seagrass traits have similar contributions to energy dissipation, except for the extreme case that seagrass grow at 1 m depth (Extended Data Fig. 8). Hence, conservation and restoration efforts should target, in particular, shallow waters, where the cover of *P. oceanica* has the greatest role on coastal protection.

The results presented here reveal a prominent role of P. *oceanica* seagrass in the defense of Balearic coastal areas. The present distribution and abundance of *P. oceanica* around the Balearic archipelago induces a reduction of the extreme total water level (eTWL) of 0.7 m, on average, both on beaches and ports. This represents, respectively, a remarkable 40% and 15% reduction relative to the TWL expected if the region would be devoid of *P. oceanica*. Hence, loss of seagrass would lead to the impacts that are now experienced every 50 years (e.g., Gloria storm) to recur every year on beaches and every 5 years on ports.

Future impacts will be aggravated due to sea level rise and the projected loss of P. *oceanica*with increased intensity and recurrence of marine heat waves^23,25^. Specifically, seagrasses loss would imply an average increase of the eTWL of 1.46 m by the end of century, with 46% of that value induced by the sea level rise and 54% due to the loss of seagrasses. Conservation of seagrass along the century would reduce the increase in eTWL to half, i.e., about 0.67 m, almost entirely due to the contribution of the sea level rise. In terms of the impacts associated to extreme storms, this means that impacts similar to those under the Gloria storm would be experienced, at the end of the century, every year on beaches and every 5 years on ports due to the sea level rise. If seagrass were completely lost, such destructive impacts would be expected several times per year on beaches and every two years on ports.

The results presented provide a compelling quantification of the fundamental role that seagrass meadows play in coastal defense currently and the importance of conserving seagrass to mitigate the impacts of sea level rise and increased storm activity on vulnerable coasts. Indeed, our main finding is that seagrass loss and sea level rise can have similar, and additive impacts in magnitude on Balearic coasts by the end of the century. With much of the GDP of the Balearic Island depending on coastal tourism, similar to other island destinations around the world, the prospect is rather adverse, particularly conservation efforts may be undermined by the vulnerability of *P. oceanica* to climate change. The Kunming-Montreal Global Biodiversity Framework pledges to stop biodiversity losses, protect 30% of ocean space and restore 30% of degraded habitats. The investments required are large, but the benefits are likely much larger, as the reduced impacts to coastal infrastructure from sea level rise and increased storms, will avoid economic losses likely far exceeding the costs of conserve and restore seagrass meadows. Deriving innovative approaches to conserve and restore P. *oceanica* seagrass meadows and increase their resistance to marine heat waves should be a priority to reduce impacts of sea level rise on coastal infrastructure and human lives.

## Methods

### Bathymetry and coastal typology

Bathymetric information is obtained primarily from the GEBCO datasets (General Bathymetric Charts of Ocean, http://www.gebco.net), provided in a 30 arc-second grid. In order to increase the spatial resolution in shallow waters, the bathymetric information is supplemented with higher resolution data from the shoreline to 40 m depth provided by the Spanish coastal authority (MITECO, https://www.miteco.gob.es/es/costas/temas/proteccion-costa/actuaciones-proteccion-costa/illes-balears/default.aspx). In this work, we consider two typologies of coastal protection (beaches and artificial structures). Beach characteristics were also derived from the MITECO data base, including information on the shape contour and granulometry of 869 beaches along the Balearic archipelago . Throughout equilibrium profile theory^33^, we obtained the beach profile considering the physical interaction between the granulometry and wave’s energy in front of the beach^31^.

We considered two types of structures that mainly protect the dock from storms to characterize the ports: a dam with a relatively gentle slope and a vertical wall. There are 34 main ports in the region (5 commercial ports belonging to the National Harbour Authority, Puertos del Estado, and 29 recreational ports managed by regional authorities, see Fig. 1). For the commercial ports we assume a toe’s depth of 20 m, which is characteristic of this kind of structures in the Mediterranean sea^34^ and for the recreational ones 5 m depth at the same location (Table SI1).

## Present marine climate

The characterization of sea level variability has been done by merging two datasets. On the one hand, we use a daily coastal sea level reconstruction based on tide gauge observations covering the whole archipelago for the period from 1980 to 2015. The reconstruction is based on an optimal interpolation of tide gauge observations which provides an accurate representation of sea level variability from daily to interannual scales along all the coastal region^35^. The product is available at https://doi.org/10.1594/PANGAEA.945345. On the other hand, the subdaily variability responsible for extreme sea level has been characterized using the shallow water Hydrostatic Padua Surface Elevation (HYPSE) model to simulate storm surges^36^. The model was forced by the atmospheric fields provided by the COSMO-ERAINT011 (12 km resolution covering from 1979 to 2013 at 3 hourly resolution^37^.

Both datasets have been validated using the observations of the tide gauges at three different locations in the Baleric Islands (in Palma, Alcudia and Mahón, denoted by TG1, TG2 and TG3 in Fig. 1, respectively). Daily sea level shows an RMSE of 0.016, 0.015 and 0.018 and a time correlation of 0.99, 0.99 and 0.98 for the tide gauges TG1, TG2 and TG3, respectively. Three hourly storm surge (removing daily sea level signal) shows a RMSE of 0.015, 0.014 and 0.014 and a time correlation of 0.67, 0.73 and 0.73 for the tide gauges TG1, TG2 and TG3, respectively. An example of the good agreement of the datasets with observations is presented in Extended Data Fig. 1, left and center panel for january to june, 2012.

Wind wave variability for the period 1979–2013 in deep waters has been characterized using a wave model simulation done with the WAM model^38^and forced by the winds from COSMO-ERAINT011^37^. The simulated 3 hourly wave data was validated using observations from two different offshore buoys (Dragonera and Mahón), with a RMSE of 0.20 m and 0.25 m, respectively and a correlation over 0.85 in both cases (see Extended Data Fig. 1, right panel ; Agulles et al., 2021).

## Future marine climate

Projections of future mean sea level include all the factors that play a role at a global scale (mass variations linked to the addition or removal of water from the ocean and thermal expansion due to ocean warming) and at regional scale (gravitational fingerprint of changes in the ocean mass, changes in the circulation patterns, mass redistribution by atmospheric pressure and wind and land motion). In this work, we use the projections described by^39^and^31^. These are mainly based on the results of^40^ which include the global and regional signals of the changes in the mass component and the dynamic contribution in the Atlantic. Under GHG scenario RCP8.5, mean sea level in the region is projected to rise between 0.50 to 1.10 m, while under GHG scenario RCP4.5 it would rise between 0.42 to 0.65 m by 2100.

Projected changes in storm surge and open ocean waves for the Balearic Islands are very small under scenarios RCP8.5 and RCP4.5. The projected changes are about ~ 0.01 m and ~ 0.1 m, for the storm surge and the extreme waves, respectively^31^. Therefore, we can consider that the extreme value characterization done for the storm surge and the wave components for the present climate will also be representative of the future climate.

## Seagrass parameters

To identify the presence of seagrass at a regional scale, we use information of the seagrass distribution provided by the Spanish Seagrass Atlas^41^, which consists on a database fed by field observations from different research and regional institutions. This database provides the location and depth of seagrass meadows around the Balearic Islands, while lacking information on changes of seagrass distribution (Extended Data Fig. 2).

Aiming at assessing the variability of the position of the seagrass shallowest front (hereafter referred as seagrass front) along the years, two databases have been used for four study sites (Fig. 1). First, we used orthophotos covering the period between 1956 and 2015 with a spatial resolution of 0.5 m from the Government of the Balearic Islands (http://ideib.caib.es/visor/). There are 4, 6, 2 and 3 valid orthophotos for PDP, BDA, CLM and SNB, respectively. Second, we used satellite images from SENTINEL 2, with a spatial resolution of 10 m and covering the period from 2015 to 2021 at monthly resolution (https://scihub.copernicus.eu/dhus/#/home). A subjective quality control has been performed to keep only those images clear enough to identify the seagrass front. Overall, the dataset includes a total of 230 images distributed as follows: 14 for PDP for the time period from 2006 to 2021, 70 at CLM from 1956 to 2021, 76 at BDA from 2002 to 2021 and 70 at SNB from 1984 to 2021.

In this work, we aim at identifying the position of the seagrass front, to obtain the minimum relative depth (water depth/plant height) which plays a role in the wave energy dissipation. Thus, instead of using automated algorithms^42,43^, the shallowest limit of the meadow is identified by visual inspection over a georeferenced image based on the color contrast, and the polyline is drawn by hand (see an example in Extended Data Fig. 3). Once defined, the corresponding average depth and standard deviation (STD) of the seagrass front, for each image, is obtained from the bathymetric data. Finally, from the analysis of all the available images, time series of the shallowest seagrass depth have been generated for the four study sites shown in Fig. 1, black squares.

Seagrass meadow density plays an important role in the wave energy dissipation (Eq. 1). We used seagrass shoot density values measured annually, from 2000 to 2012^9^,, see Fig. 1 green dots, for locations and Extended Data Table S1 for values. Shoot density counts were conducted by divers in permanent representative plots (between 0.06 m^2^ and 0.25 m^2^) mostly in summer.

## Numerical modelling system

Total water level (TWL) at the coast can be represented as a combination of several components: the mean sea level, the storm surge and the wave runup. The two first components can be faithfully represented by the databases presented above. However, the wave runup at the coast can largely differ from the open sea waves provided by the above-mentioned regional models. In consequence, a numerical modelling system able to simulate the wave runup has been implemented. The system is composed by nesting two different numerical models. Firstly, we use a model able to propagate open sea waves up to nearshore and secondly, a model able to simulate the wave runup at the coast in the presence of submerged vegetation.

## Nearshore maritime climate

Physical processes as refraction, diffraction, white-capping, bottom friction and breaking, among others, modify the waves parameters on their way from deep to shallow waters^44^. Therefore, the wave fields simulated in deep waters need to be transferred up to the nearshore. To do so, we implement a hybrid downscaling methodology using a clustering algorithm^45^and the SWAN numerical model^46^to propagate the historical deep waves to shallow waters (for more details see^31^. As a result, we obtain a 3 hourly nearshore wave reconstruction for the period (1979–2013) covering the whole Balearic Islands with a spatial resolution of 200 m.

## Wave runup in presence of submerged vegetation

The most important transformation of wave characteristics occurs in the shoaling and swash zone, where diffraction, shoaling and breaking highly reduce the waves energy^47^. Moreover, in this critical zone, the bottom friction plays a paramount role in the energy wave dissipation, especially over vegetated grounds^6,48,49^.

In order to simulate these processes, the XBEACH model has been implemented^18^. The model solves phase-averaged coupled 2D horizontal equations for wave propagation, flow, sediment transport, and bottom changes, that allows, among other parameters, to obtain the wave runup at the swash zone^31^. Additionally, we consider the vegetation module of XBEACH, which includes wave damping and wave breaking over vegetation fields at variable depths. Based on a nonlinear formulation of the drag force, either irregular or monochromatic waves can be modelled considering geometric and physical characteristics of the vegetation field^16,17^ where the time-averaged energy dissipation through a vegetation field can be expressed with the following equation:

1 $$\left\langle {\varepsilon_{v} } \right\rangle = \frac{1}{2\sqrt \pi }\rho C_{D} b_{v} N\left( {\frac{kg}{{2\sigma }}} \right)^{3} \frac{{sinh^{3} \left( {k\alpha h} \right) + 3sinh\left( {k\alpha h} \right)}}{{3kcosh^{3} \left( {kh} \right)}}H_{rms}^{3}$$

where ${b}_{v}$ is the vegetation stem diameter, $N$ is the vegetation density, $k$ is the wave number, g is the gravitational acceleration, $\sigma$ is the wave frequency, $h$ is the water depth, ${C}_{D}$ is the drag coefficient and ${H}_{rms}$ is the root mean square wave height.

Four sensitivity runs have been performed forcing the 1D version of XBEACH with observed nearshore waves and changing the damping coefficient due to wave-seagrass interaction (Extended Table 1). In order to calibrate the vegetation module, the results have been compared with observed swash (back-and-forth motion of water onto the shore after waves breaking), retrieved from timestack images from 2012 to 2018, provided by the Balearic Islands Coastal Observing and Forecasting Facility (SOCIB)^50^, in PDP and CLM beaches and post-processed by^51^. The results show that the inclusion of the vegetation module improves the results in both beaches (see Extended Table 1 and Extended Fig. 4). Without vegetation, the correlation is 0.79 (0.64) and the RMSE 0.19 m (0.15 m) for PDP (CLM). With the optimal drag coefficient (Test 3), the correlation is almost the same 0.80 (0.63) but the RMSE is strongly reduced to 0.08 m (0.13 m).

Once the vegetation module has been calibrated an additional validation of the model has been carried out using waves observations from another field experiment^13^. In that experiment, the wave H_rmse_was measured along a cross-shore transect in CLM beach during the period from 7 to 23 July 2009 (Extended Data Fig. 9). In that beach, seagrass was present up to a depth of 6 m and the nearshore wave height observed during the experiment ranged from 0.3 to 1.2 m when a moderate storm hit the area. The wave propagation during the same period was simulated using XBEACH with and without considering the presence of seagrass. In this case, the drag coefficient is set to Cd = 0.05 but we consider a leaf length (Av) of 0.80 m, according to the seagrass characteristics reported for that location during the experiment^13^. The results show that, at the deepest location (~ 17 m depth), there are no differences in the simulation without and with seagrass, with an RMSE of 0.23 and 0.22 m, respectively (P1 in Extended Fig. 9). However, as the wave steepness (H_rmse_/depth) increases closer to the coast, the simulation with the vegetation module shows better skills, with a RMSE reduction at the shallower depth (P4 in Extended Fig. 9) from 0.27 m to 0.11 m.

Additional experiments have been performed to evaluate the relative importance of different vegetation parameters (minimum depth of seagrass, leaf length, stem diameter and the shoot density). For the Cala Millor beach we have computed the runup under mean and extreme wave conditions changing each time one of the vegetation parameters (Extended Fig. 8). Those sensitivity experiments have shown that the impacts are additive and that what most influences wave dissipation is the presence/absence of seagrass between 1 and 3 m depth.

## Look-up tables

Running XBEACH for all locations (869 beaches and 34 ports) and for the whole period of the study (1979–2014) is computationally too expensive. Therefore, to obtain a high-resolution wave runup (3 hourly time series), we use a cost-effective methodology already tested by^32^. In particular, 39 sea states (i.e., pairs of Hs-Tp) that encompass all range of the nearshore wave climate obtained by the SWAN model simulation are defined, see Sect. 3.1 of the referenced paper. Then, XBEACH is used to propagate, from intermediate depths up to the swash zone, these 39 sea states under different conditions of bathymetry, seagrass coverage and seagrass abundance to generate several look-up tables. These tables allow to directly link nearshore sea states with the wave characteristics at the coast without the need of running the model again. The look-up tables are thus used to transform 3-hourly time series of nearshore waves for present climate (1979–2014) to Hs at the dikes toe or to wave runup at the beach, using the look-up table that corresponds to the local bathymetry and seagrass characteristics (see a sketch of the procedure in Extended Data Fig. 5).

In this process, we have considered 11 bottom equilibrium profiles, 5 scenarios of seagrass coverage and two meadow densities. The beach profiles correspond to different granulometries of sand beaches (from a grain size of 0.015 to 0.075) and have been obtained based on the formulation of^52^. The scenarios of seagrass coverage considered are (1) no presence of seagrass, (2), seagrass up to the shoreline, (3) up to 2 m depth, (4) up to 5 m depth and (5) up to 7 m depth. The meadow densities considered are 615 shoots/m^2^ and 200 shoots/m^2^, which are representative of a well-preserved meadow and a deteriorated one^9^, respectively. Overall, we have performed 4290 (39 × 11 × 5 × 2) simulations of XBEACH that covers all possible combinations of sea states, beach profiles and seagrass coverage in the region (Extended Data Fig. 5).

XBEACH explicitly, provides the wave runup on the beaches but it has not been created originally for coastal dikes or seawalls. For those cases, the common approach is to use empirical formulation based on laboratory and field controlled experiments^53^. For dikes with a relatively gentle slope (28 out of 34 in our case, see Table SI1) the wave runup is estimated as follows (Eq. 1):

2 $$RU_{2\% } = H_{mo} *1.65*{\varvec{\gamma}}_{b} *{\varvec{\gamma}}_{f} *{\varvec{\gamma}}_{\beta } *{\upvarepsilon }_{m - 1,0}$$

where ${H}_{mo}$ is the wave height at dike’s toe estimated from the XBEACH simulations, and ${{\varvec{\gamma}}}_{b}$, ${{\varvec{\gamma}}}_{f}$, ${{\varvec{\gamma}}}_{\beta }$ and ${\upvarepsilon }_{m-\text{1,0}}$ are parameters that represents the berm characteristics, roughness, wave attack and breaker parameter, respectively. In our case, we consider a dike with relatively gentle slope as a design approach, with the simplest parametrization. That is, no berm (${{\varvec{\gamma}}}_{b}$=1), equivalent roughness of 0.4 (rocks with two layers and permeable core), perpendicular wave attack against structure (${{\varvec{\gamma}}}_{\beta }=1$) and a breaker parameter equal to 3, which is the typical configuration of Mediterranean coastal structures used to design harbors^34^. For those ports that have been designed with a vertical wall as the main protection structure (6 out of 34), we use another approximation showed also by Eurotop 2018 manual, (Eq. 2):

3 $$RU_{2\% } = H_{mo} *1.8$$

## Scenarios of total water level evolution

In order to produce future scenarios of TWL at the beaches or ports, we need projections of future sea level, waves and seagrass characteristics. Unfortunately, it is difficult to provide robust projections for the future of the *P. oceanica*in the coming decades. Whereas its resilience against the increase of the sea temperature and marine heat waves largely depends on populations thermal threshold^25,54^, which are quite well established for the Balearic Islands, there is a lack of information about its vulnerability to severe storms, despite evidence that they can trigger *P. oceanica*losses^55,56^. Also, anthropogenic factors (e.g., pollution, anchoring) can lead to losses.

An analysis of the available data^9^ shows that, for seagrass meadow shallower than 10 m, the average shoot density ranged from 300 to 1000 shoot/m^2^, with large interannual variability ( temporal std of 103 shoot/m^2^), and a an underlying decline rate at −8.8 ± 2.1 shoot/m^2^ /yr . Mean shoot density of meadows deeper than 10 m was lower, ranging from 100 to 500 shoot/m^2^ and with less interannual variability (70 shoot/m^2^). However, deep meadows showed a stronger negative trend at −17 ± 1.2 shoot/m^2^/yr. In other words, the rate of the meadow degradation below 10 m doubles the rate observed in shallower waters. It is dangerous to extrapolate those trends for the next decades as far as the dynamics of the meadows and the impact of climate change may be strongly non-linear, but if they would hold, by the end of this century coastal areas in the region would be devoid of *P. oceanica*.

Therefore, we have opted to define three scenarios for the seagrass evolution, starting with observed seagrass density to consider that the meadows (1) remain in steady state, (2) disappear by the end of the century due to global warming, and (3) an intermediate scenario where the meadows are progressively degraded from 615 shoots m^−2^ to 200 shoots m^−2^ by end of century. This last scenario would represent the adoption of strong measures to reduce the seagrass decline (e.g. with replanting or with new protection policies) that may lead to a moderate decline.

In summary, five numeric experiments were performed (Table 2). Two of them are devoted to the analysis of the role of seagrass on coastal protection under present climate conditions (PRES_NS and PRES_WS). The other three focus on the future evolution of TWL, assuming sea level evolves as it is projected under the RCP8.5 GHG scenario but with different scenarios for seagrass evolution (FUT_NS, FUT_615 and FUT_200).

**Table 2:** Numerical experiments performed for different time periods and under different scenarios of seagrass evolution.

Scenarios of TWL	Time coverage	No Seagrass	Well Preserved Seagrass (615sh/m^2^)	Degraded Seagrass (200sh/m^2^)
PRES_NS	1979–2014	X		
PRES_WS	1979–2014		X	
FUT_NS	2015–2100	X		
FUT_615	2015–2100		X	
FUT_200	2015–2100			X

## Supplementary Information

Supplementary Information 1.

Supplementary Information 2.

Supplementary Information 3.

Supplementary Information 4.

Supplementary Information 5.

Supplementary Information 6.

Supplementary Information 7.

Supplementary Information 8.

Supplementary Information 9.

Supplementary Information 10.

Supplementary Information 11.

## Data Availability

Data inputs that are required to reproduce the experiments in this study and the main experiment outputs are freely available on Zenodo at https://doi.org/10.5281/zenodo.10657960. And also provided as supplementary information file.
