# Supplementary figures and images for: Mediterranean seagrasses provide essential coastal protection under climate change
Source: Sci Rep. 2024 Dec 4;14:30269. doi: 10.1038/s41598-024-81026-5 (PMC11618301; doi:10.1038/s41598-024-81026-5)

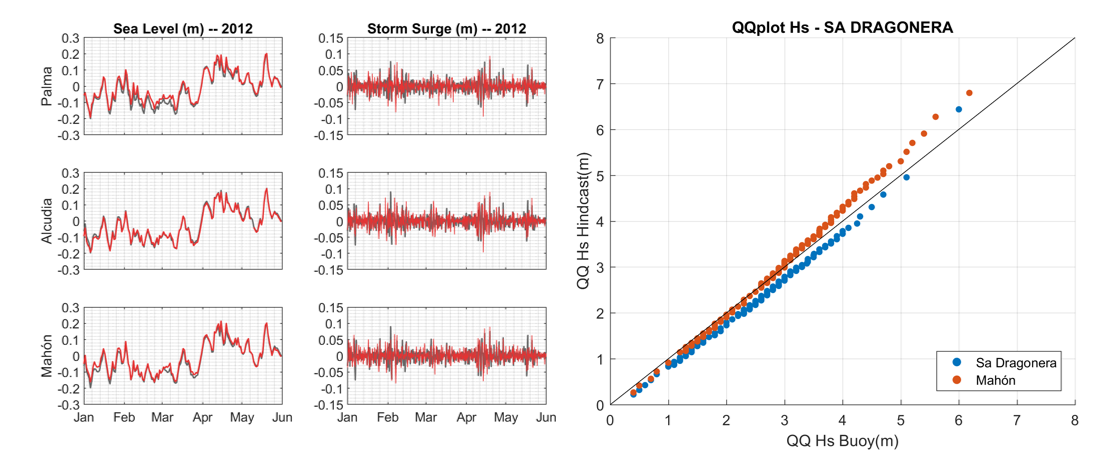

Supplement: Supplementary file 2 — Supplementary Information 2. [file 41598_2024_81026_MOESM2_ESM.tif]

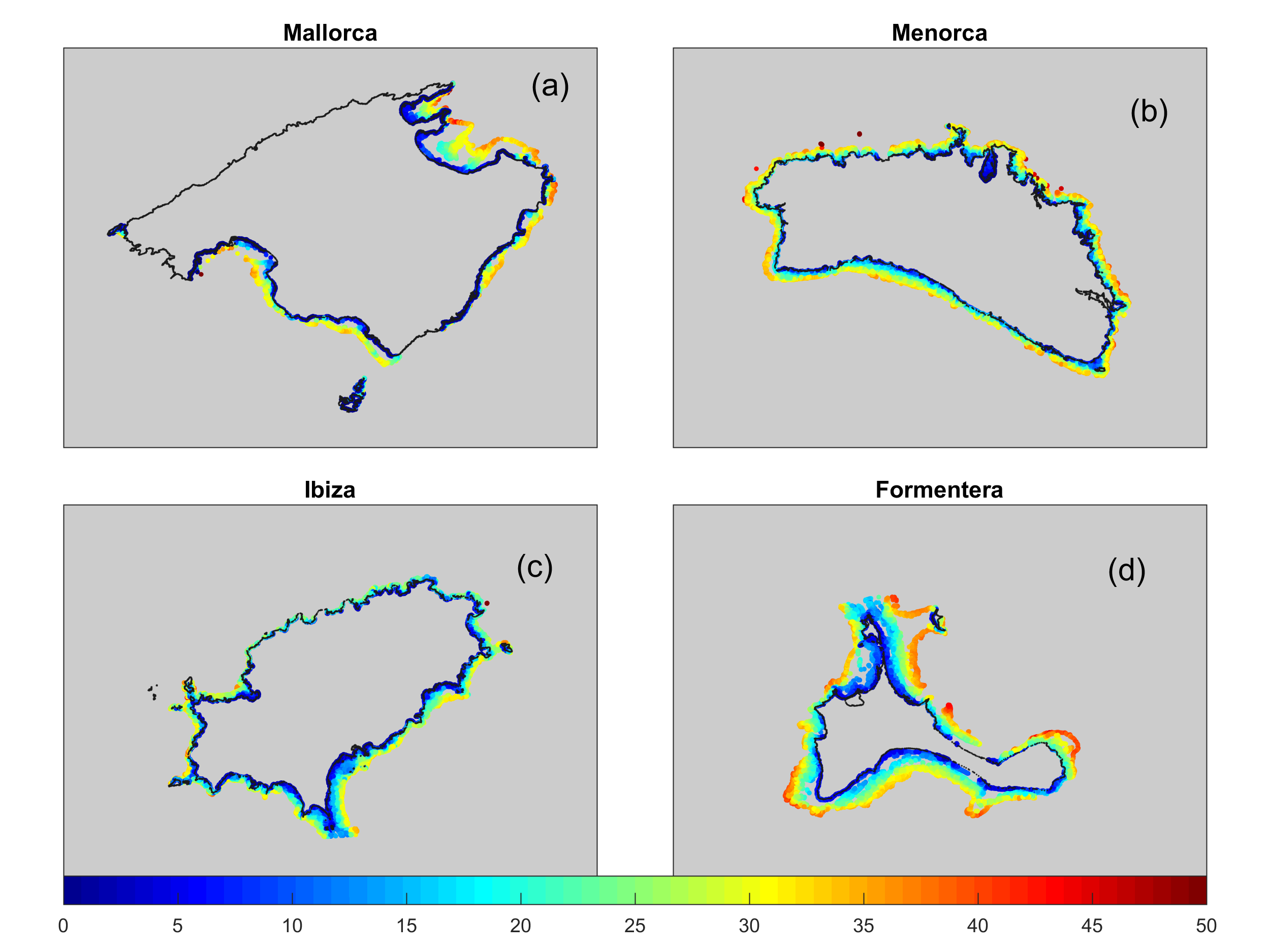

Supplement: Supplementary file 3 — Supplementary Information 3. [file 41598_2024_81026_MOESM3_ESM.tiff]

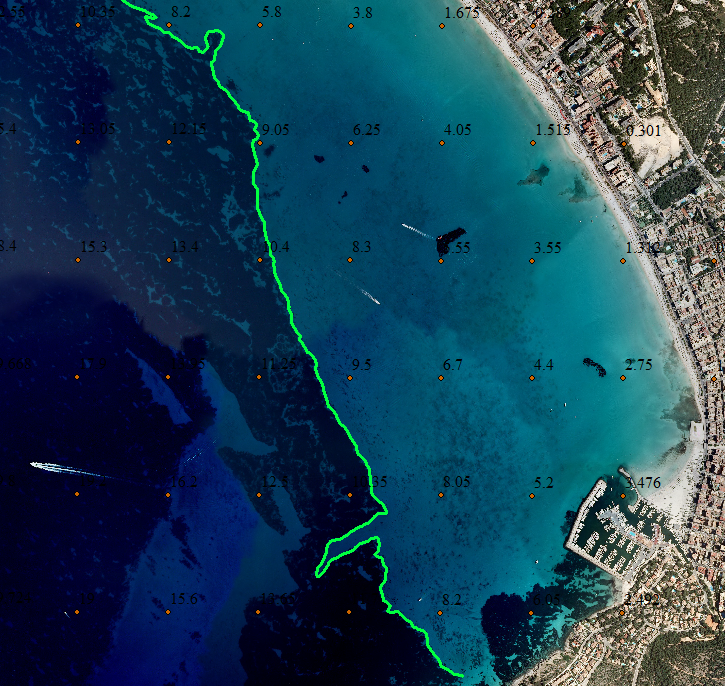

Supplement: Supplementary file 4 — Supplementary Information 4. [file 41598_2024_81026_MOESM4_ESM.tif]

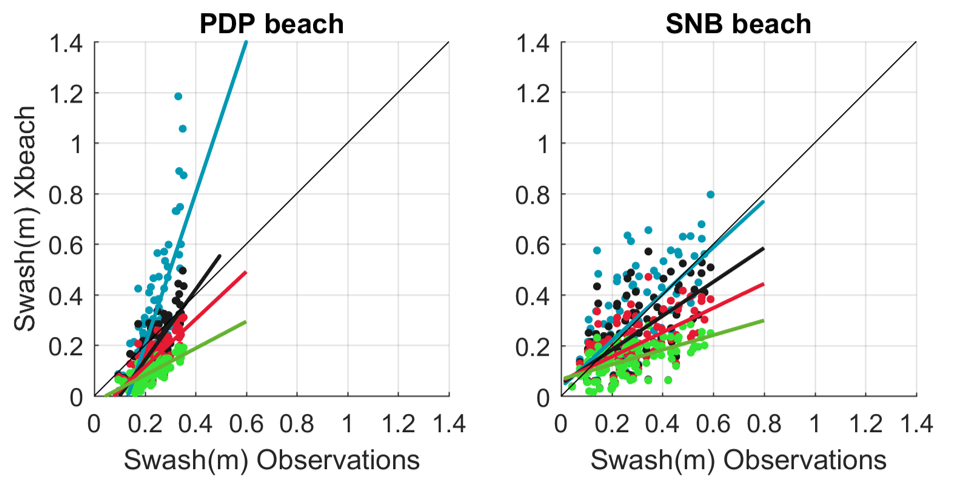

Supplement: Supplementary file 5 — Supplementary Information 5. [file 41598_2024_81026_MOESM5_ESM.tif]

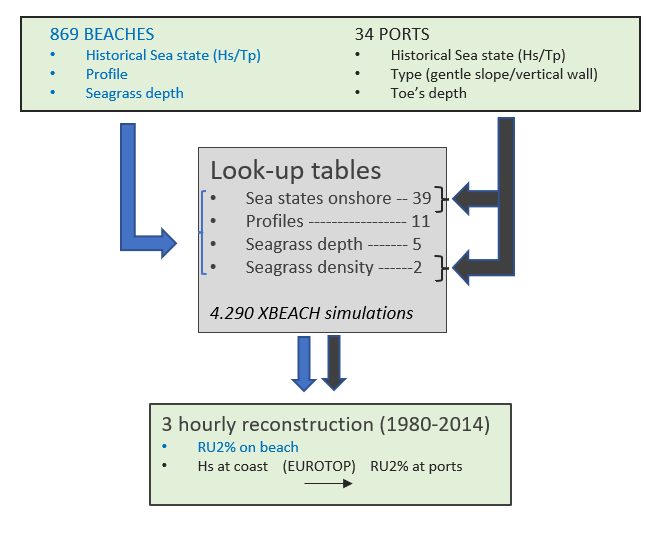

Supplement: Supplementary file 6 — Supplementary Information 6. [file 41598_2024_81026_MOESM6_ESM.tif]

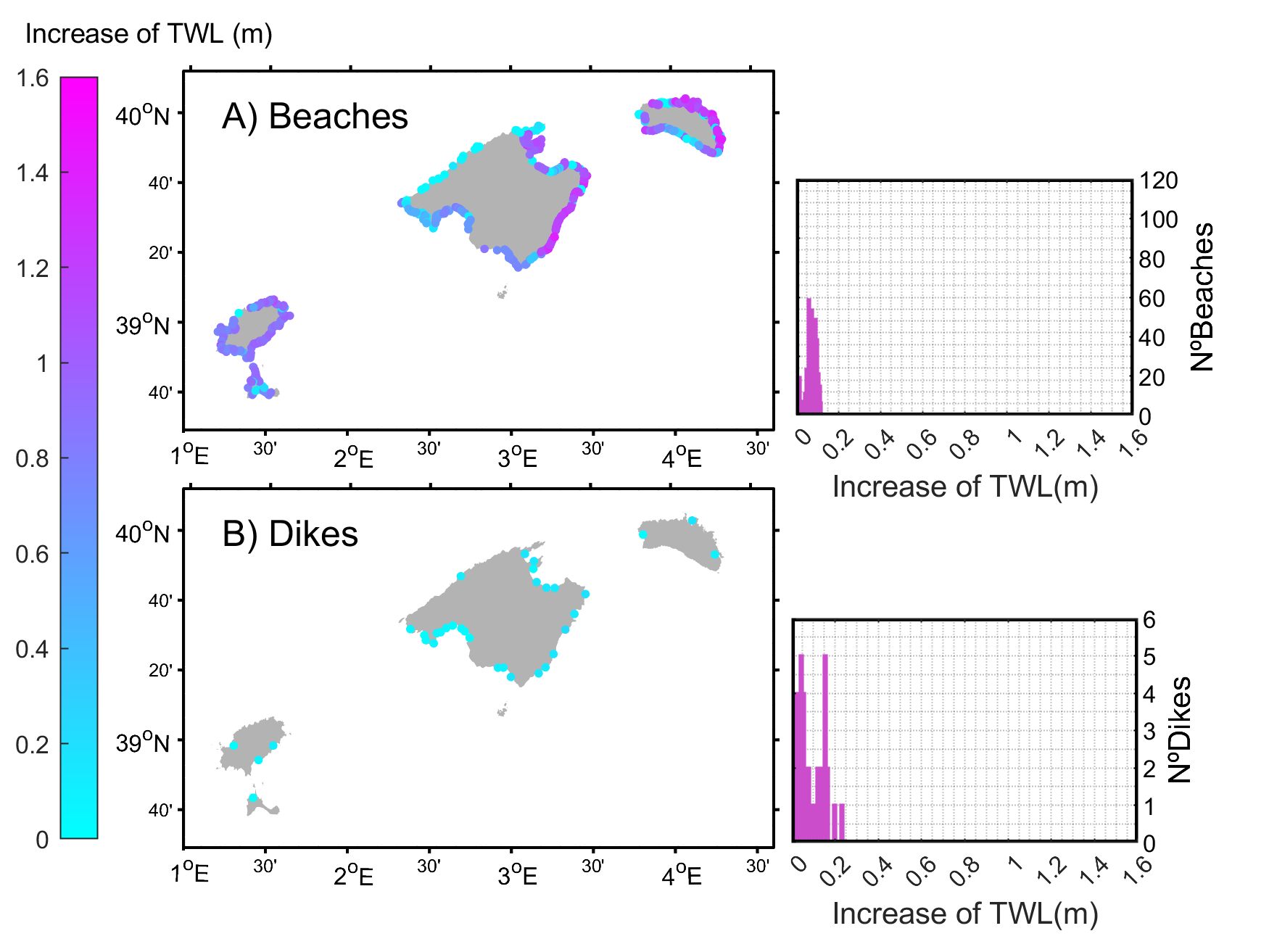

Supplement: Supplementary file 7 — Supplementary Information 7. [file 41598_2024_81026_MOESM7_ESM.tiff]

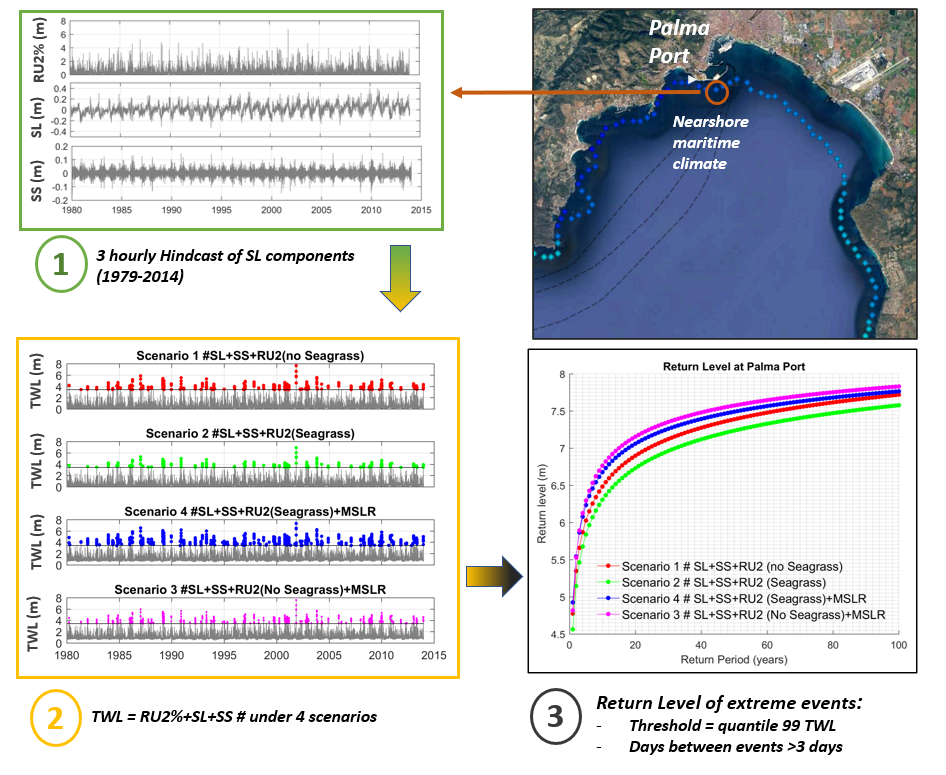

Supplement: Supplementary file 8 — Supplementary Information 8. [file 41598_2024_81026_MOESM8_ESM.tif]

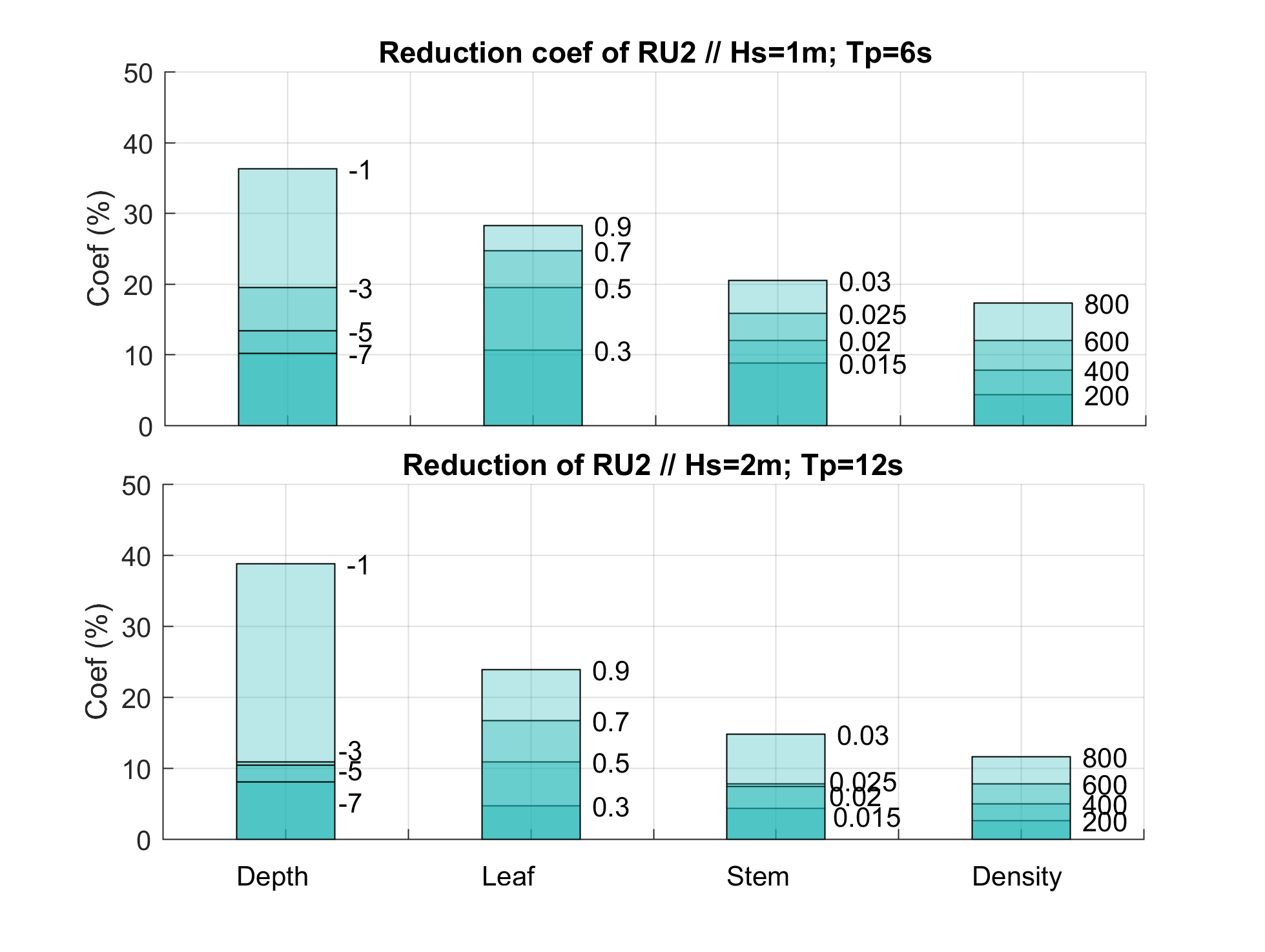

Supplement: Supplementary file 9 — Supplementary Information 9. [file 41598_2024_81026_MOESM9_ESM.tiff]

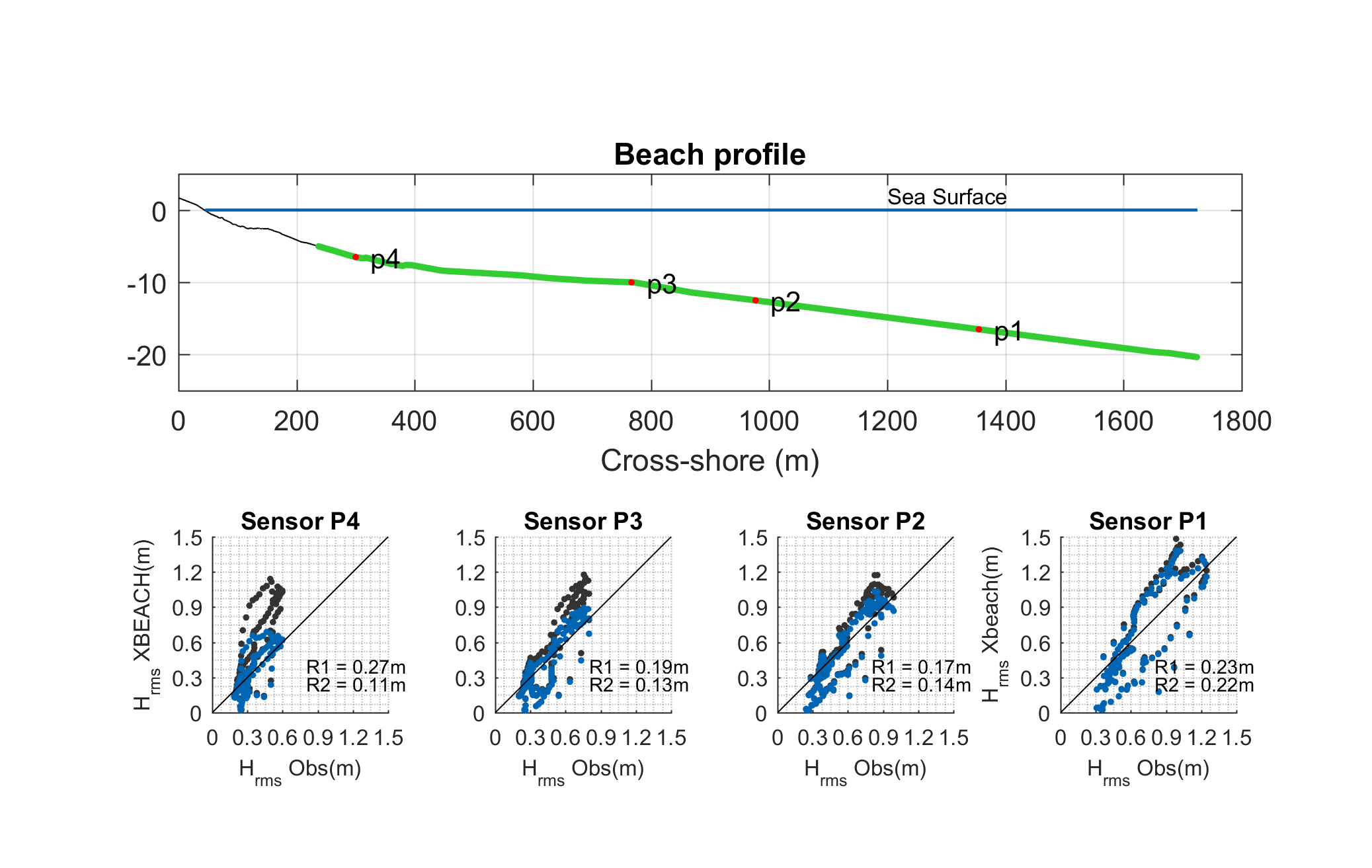

Supplement: Supplementary file 10 — Supplementary Information 10. [file 41598_2024_81026_MOESM10_ESM.tiff]
