# Supplementary material for: Mediterranean seagrasses provide essential coastal protection under climate change
Source: Sci Rep. 2024 Dec 4;14:30269. doi: 10.1038/s41598-024-81026-5 (PMC11618301; doi:10.1038/s41598-024-81026-5)
Supplement: Supplementary file 11 — Supplementary Information 11. [file 41598_2024_81026_MOESM11_ESM.docx]

## **Extended data**

| **Tests** | **Ah (m)** | **Bv (m)** | **N (shoots/m2)** | **Cd** | **RMSE (m)** | | **Correlation** | |
| --- | --- | --- | --- | --- | --- | --- | --- | --- |
|  |  |  |  |  | **PDP** | **CLM** | **PDP** | **CLM** |
| Test 0 | - | - | - | - | 0.19 | 0.15 | 0.79 | 0.64 |
| Test 1 | 0.35 | 0.02 | 615 | 0.20 | 0.13 | 0.17 | 0.79 | 0.59 |
| Test 2 | 0.35 | 0.02 | 615 | 0.10 | 0.10 | 0.13 | 0.78 | 0.61 |
| Test 3 | 0.35 | 0.02 | 615 | 0.05 | 0.08 | 0.13 | 0.80 | 0.63 |

Extended Table. 1 Parameters used in the sensitivity tests for the calibration of the drag coefficient calibration and results of the validation in terms of RMSE (in m) and correlation. The parameters considered are the mean shoot length (Ah), stem width (Bv), vegetation density (N) and drag coefficient (Cd). The symbol (-) indicates that no vegetation module has been included in the XBEACH simulations.


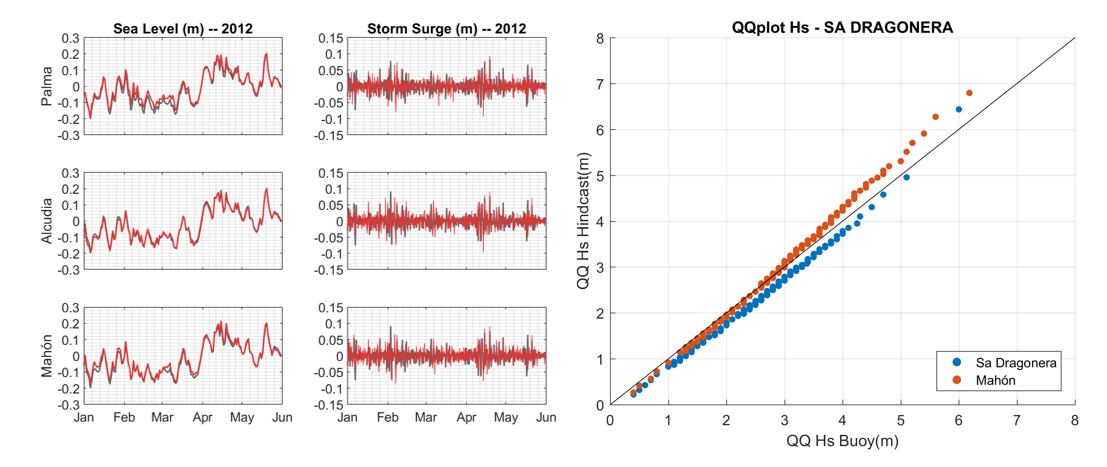


Extended Fig. 1 Validation of the sea level (left panels) and storm surge (central panels) at the port of Palma (first row), Alcudia (middle row) and Mahón (bottom row). Sea level observations are represented in grey, sea level reconstruction (in red, left panel) and simulation of storm surge (in red, right panel). On the right subplot, a quantile-quantile plot of observed and simulated significant wave height (m) by Era Cosmo at Sa Dragonera buoy (blue dots) and at Mahón buoy (red dots) is showed.


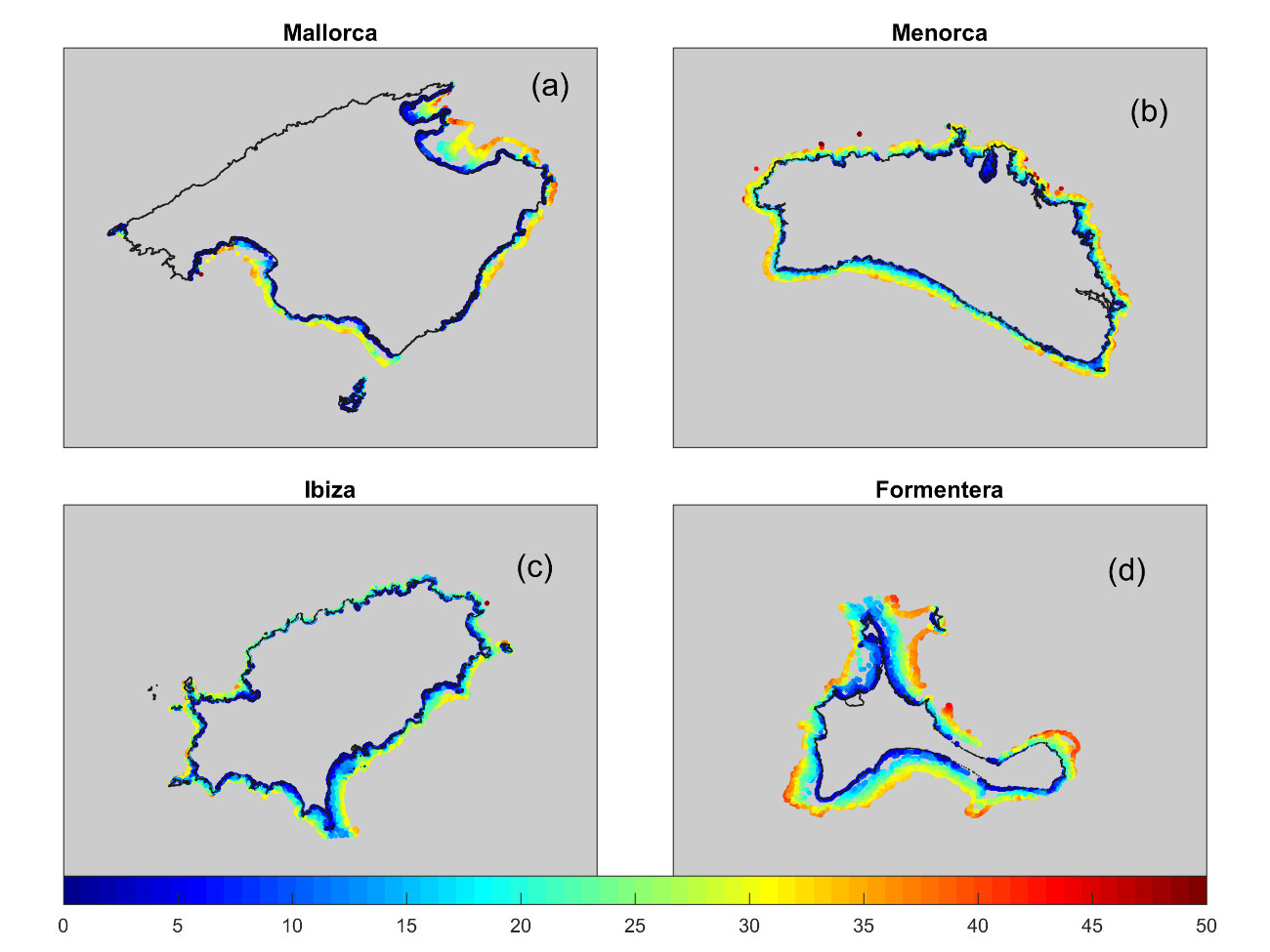


Extended Fig. 2 Seagrass meadows location and depth in meters, represented by the colorbar, for the four Islands of the archipelago. Mallorca (a), Menorca (b), Ibiza (c) and Formentera (d). Figure created by Matlab version 2022b (https://es.mathworks.com/products/new_products/release2022b.html).


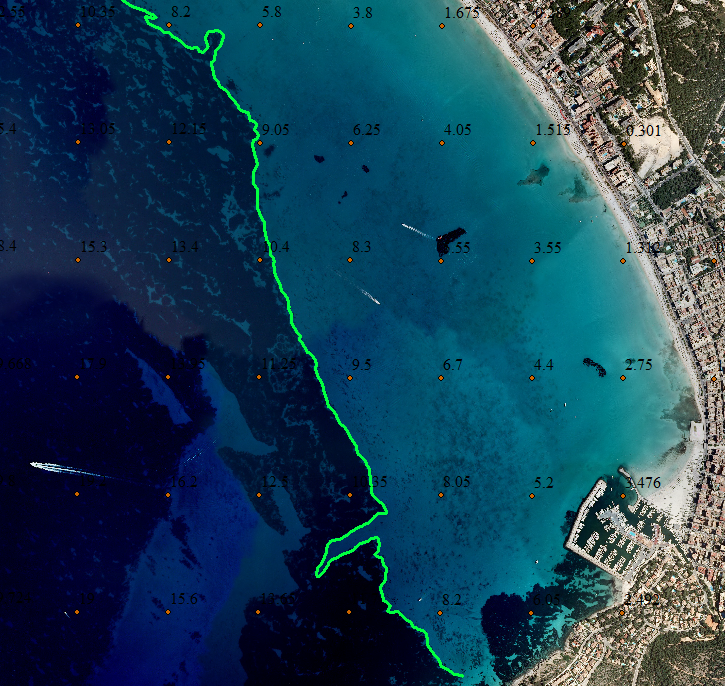


Extended Fig. 3 Example of the polyline defining the upper limit of the seagrass meadow (green line), obtained for Platja de Palma in 2008. Figure created by Matlab version 2022b (https://es.mathworks.com/products/new_products/release2022b.html).


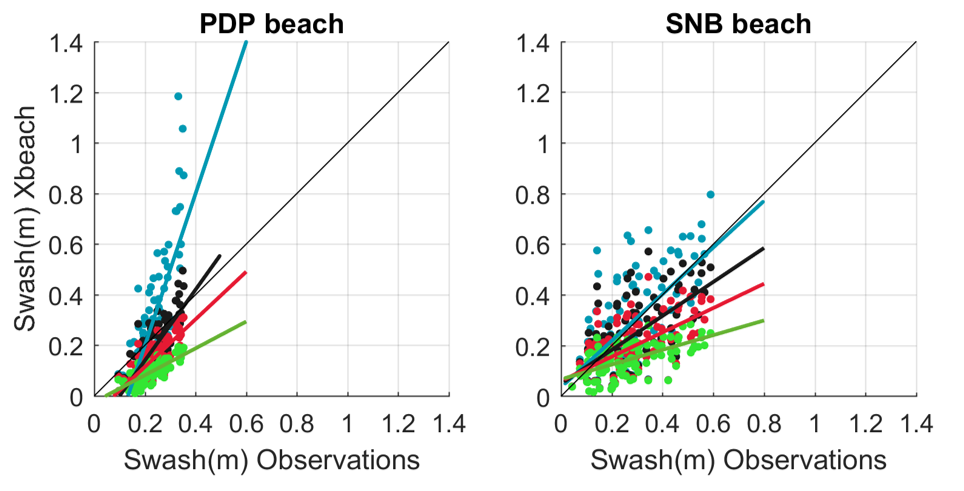


Extended Fig. 4 Results of the calibration tests for the vegetation module in PDP beach (left panel) and SNB beach (right panel). Absence of seagrass (blue), with seagrass drag of 0.20 (green), 0.10 (red) and 0.05 (black).


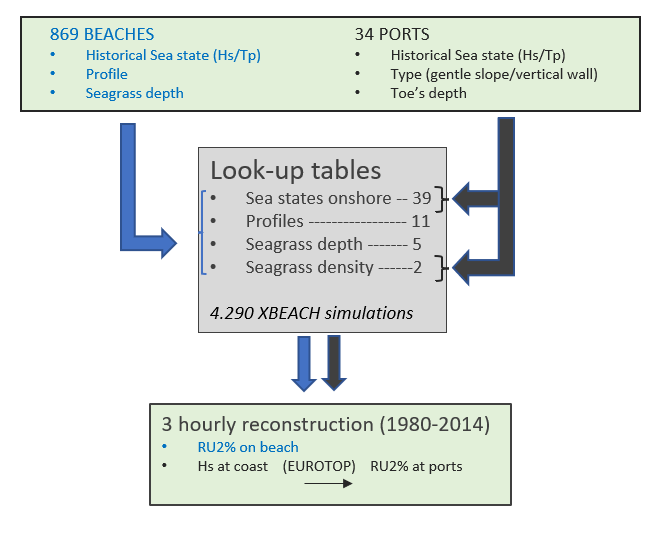


Extended Fig. 5 Diagram that describe the link between nearshore sea states with wave runup at coast depending on the coastal typology. Left path, for beaches (in blue) and right path, for ports (in black).


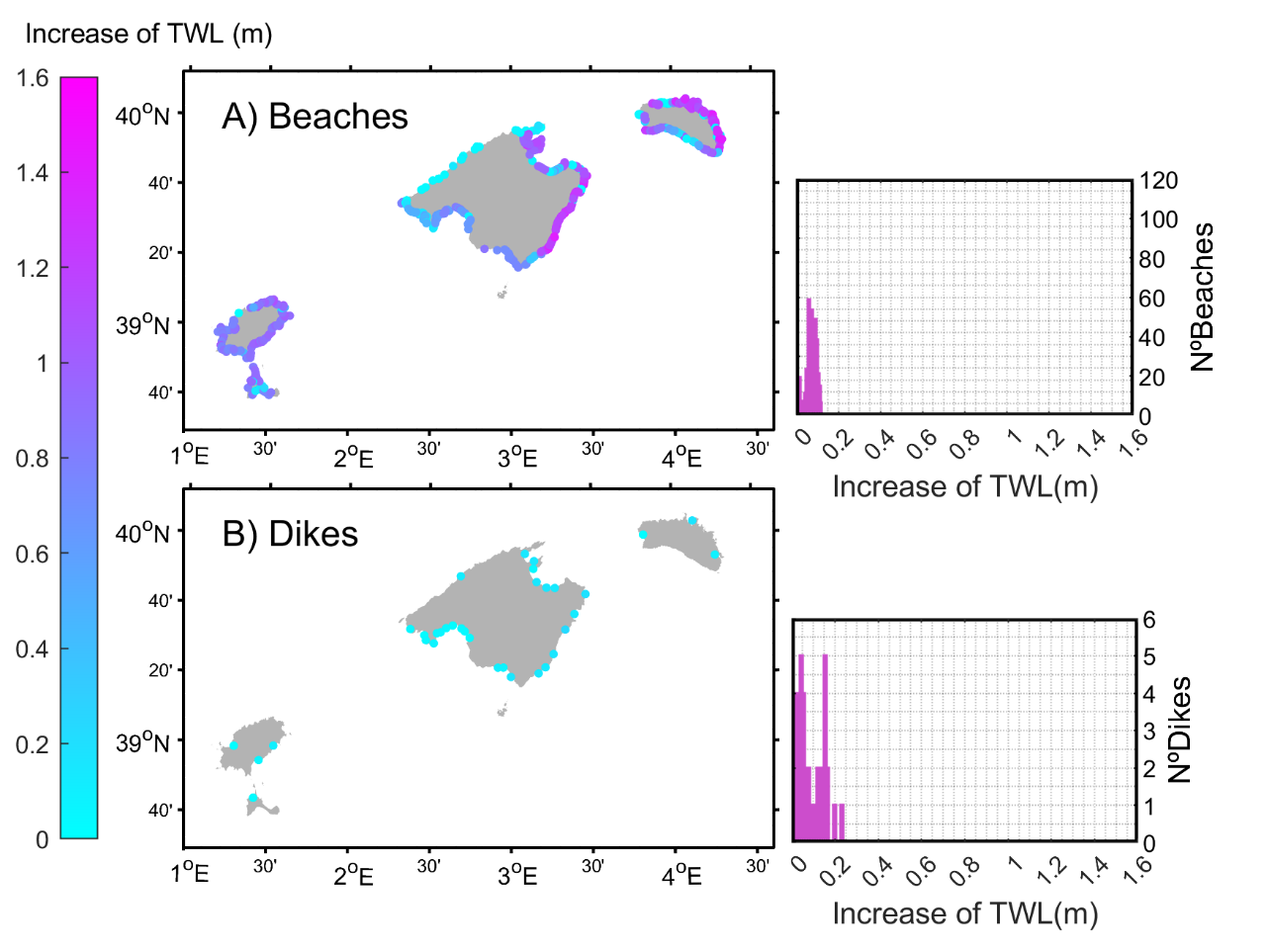


Extended Fig. 6 Increase of TWL under mean conditions in the absence of seagrasses (difference between experiment PRES_NS and experiment PRES_WS). The results are presented for the beaches (top panels) and dikes (bottom panels). The histograms of the values mapped are presented in the right side of the panels. Figure created by M_Map tool, Matlab version 2022b (https://es.mathworks.com/products/new_products/release2022b.html).


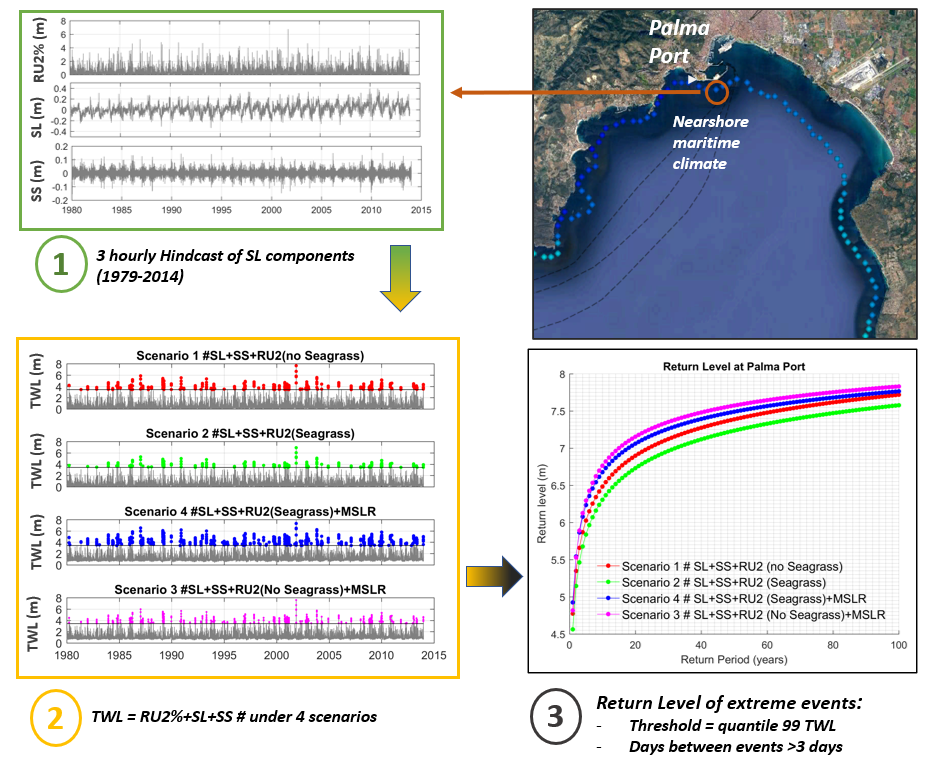


Extended Fig. 7 Diagram that represent the methodology followed to calculate Returns Periods under different scenarios considered in the manuscript. Figure created by, Matlab version 2022b (<https://es.mathworks.com/products/new_products/release2022b.html>), Google Earth Pro (<https://www.google.com/intl/es/earth/about/versions/>) and Power Point (https://www.microsoft.com/es-es/microsoft-365/buy/compare-all-microsoft-365-products-b).


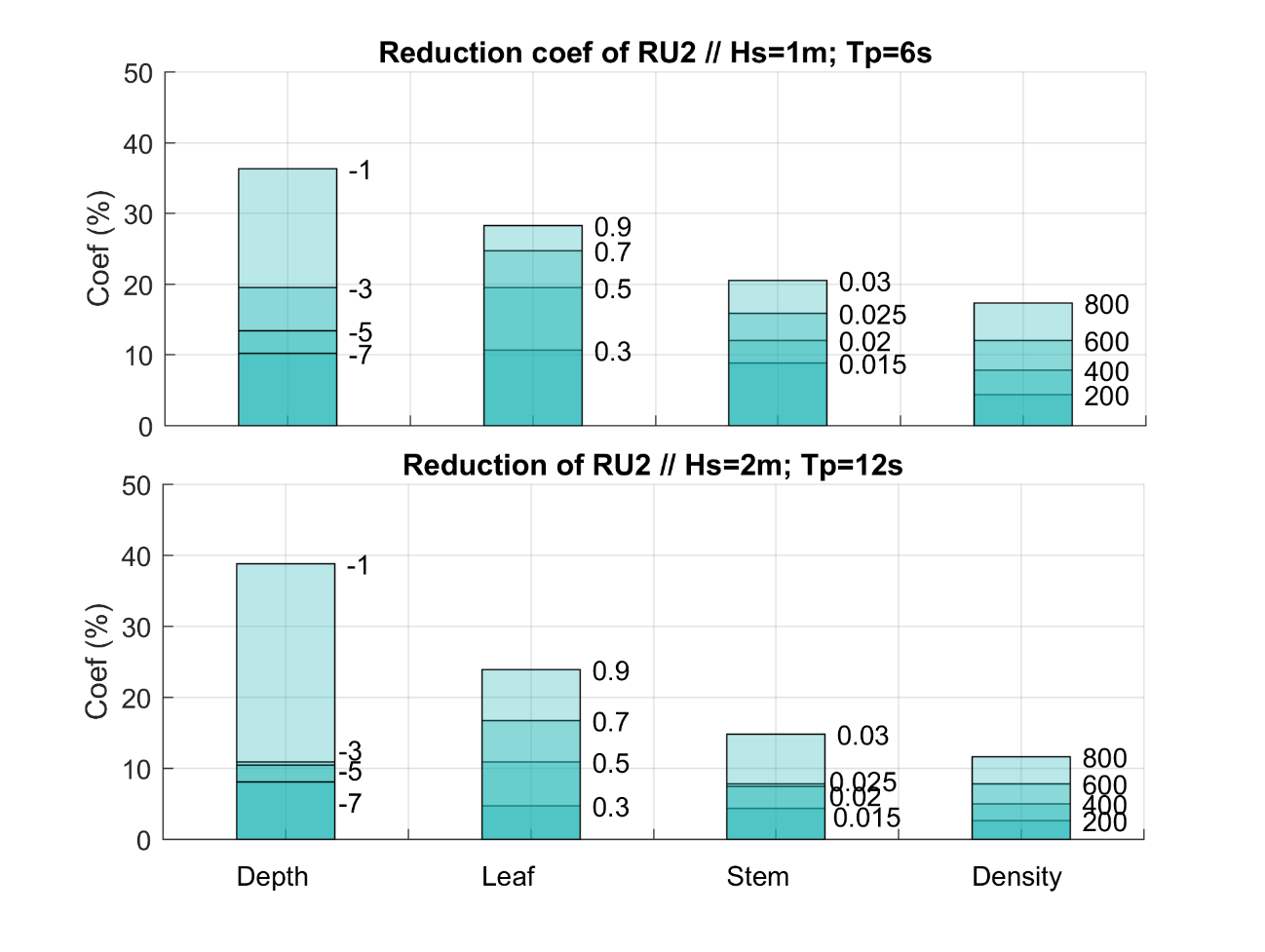


Extended Fig. 8 Sensitivity experiments of wave runup reduction in the Cala Millor Beach with different characteristics of the seagrass meadow with respect to the case of not having any seagrass. First column, minimum depth of seagrass in meters. Second column, leaf in meters. Third column, stem diameter in meters. Fourth column, the shoot density in (shoot/m^2^).


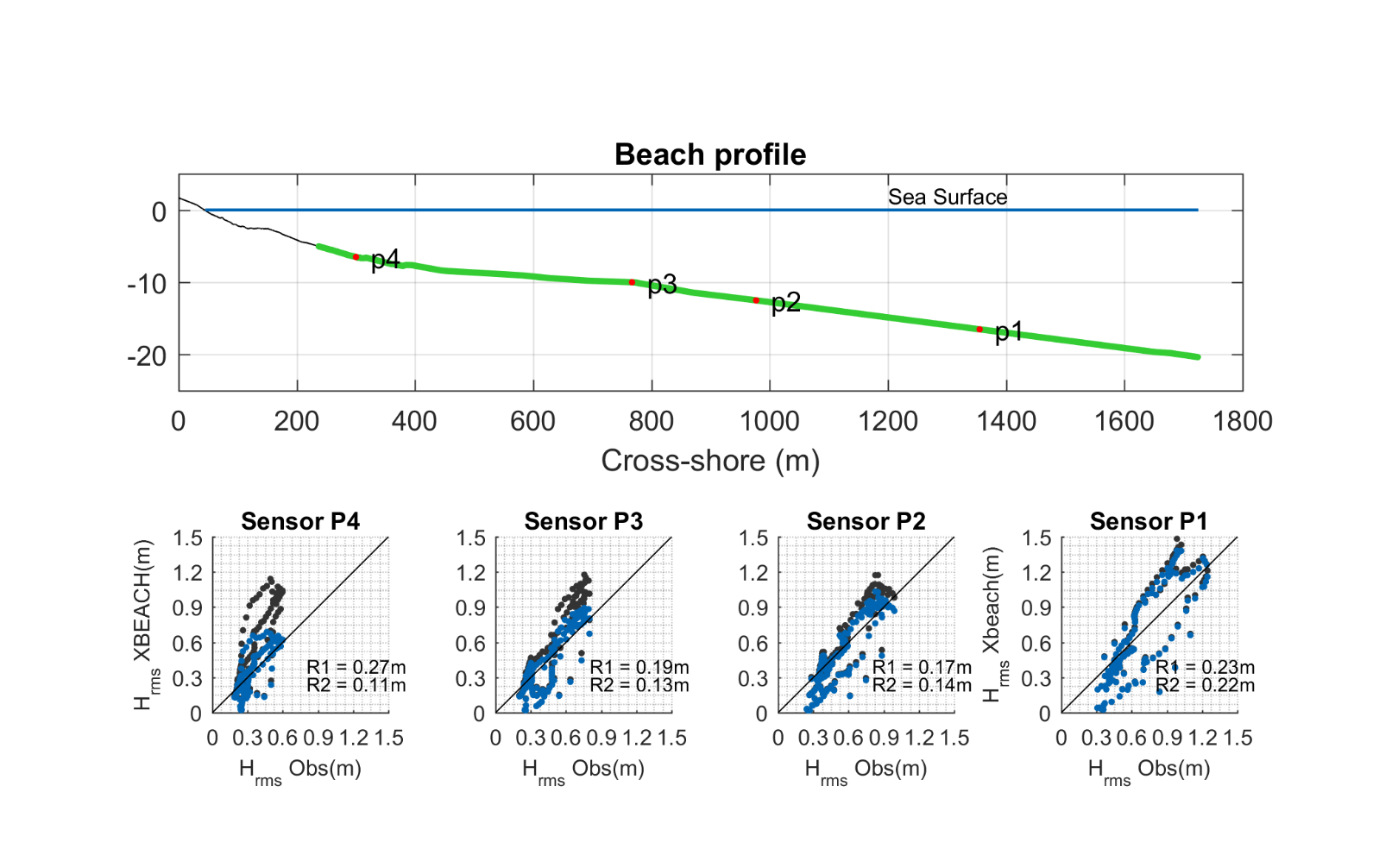


Extended Fig. 9 Validation of the XBEACH model outputs in Cala Millor along a cross-shore transect (upper panel). The location of the wave observations is denoted by the red dots (P1 to P4). The lower panels show the comparison of observed and modelled H_rms_ (in m) for the four observation points. The corresponding RMSE (in m) is included in the insets for the simulation without the vegetation module (R1, corresponding to the black dots) and with the calibrated vegetation module (R2, corresponding to the blue dots).
